# The association between blood, urine, respiratory, neurobehavioral parameters and occupational exposure to organophosphorus pesticides: a cross-sectional study among formulators

**DOI:** 10.4314/ahs.v22i3.73

**Published:** 2022-09

**Authors:** Mohammad Amin Rashidi, Hassan Asilian Mahabadi, Ali Khavanin, Leila Tajik

**Affiliations:** 1 Student Research Committee, Department of Occupational Health and Safety, School of Public Health and Safety, Shahid Beheshti University of Medical Sciences, Tehran, Iran; 2 Department of Occupational Health Engineering, Faculty of Medical Sciences, Tarbiat Modares University, Tehran, Iran; 3 Department of Occupational Health and Safety at Work Engineering, School of Health and Nutrition, Nutritional Health Research Center, Lorestan University of Medical Sciences, Khorramabad, Iran

**Keywords:** Pesticides, Organophosphorus Compounds, Blood, Urine, Lung

## Abstract

**Background:**

Organophosphate (OP) pesticides are one of the most extensively used chemical compounds all over the world.

**Objective:**

The aim of this study is to determine whether occupational exposure of the formulators to the OP pesticides, under normal working conditions, is associated with any hematotoxic, hepatotoxic, nephrotoxic, neurotoxic, and respirotoxic responses among them.

**Methods:**

28 OP formulation plant workers and 17 office workers participated in this cross-sectional study as the exposed and control groups, respectively. Blood and urine samples were collected to measure hematological, biochemical, and urinalysis parameters. American thoracic society questionnaire and spirometry tests were employed to assess the function of their respiratory system. Q16 questionnaire was also used to investigate the prevalence of neurobehavioral symptoms. The data were analyzed by SPSS v.22 software using Kolmogorov-Smirnov, T-test, Mann-Whitney U, Chi-square, Fisher, Pearson, and Spearman tests.

**Results:**

No statistically significant difference was found in hematological, biochemical, urinalysis (except in specific gravity), spirometry parameters, as well as respiratory and neurobehavioral symptoms between the exposed and the control groups. For the exposed group, however, the means of spirometry parameters were significantly lower among the smokers.

**Conclusions:**

In this study, the expected adverse health effects due to exposure to OP pesticides were not observed among the formulators; however, the risk of developing respiratory dysfunction was found to be more considerable among smoker subjects than the non-smoker ones. Further investigations are required to determine whether formulators' occupational exposures to OP pesticides result in certain adverse health effects.

## Introduction

The term pests include plants, animals and microorganisms which are harmful or dangerous to crops, livestock and particularly human health. Applying pesticides is one of the most common and to some extent, the most efficient method for pest control worldwide. Nevertheless, the strategy of using pesticides has never escaped criticism as it may leave acute and chronic adverse effects on human health and environment 1, 2. In several studies conducted in developing countries, the possible hazards of using pesticides have been reported as a major concern. Besides, the highest proportion of pesticide-related exposures, poisonings and deaths occur in these countries^1^,^3^.

Based on the official reports, in Iran, the annual consumption of pesticides is about 20–25 thousand tons (0.5% global consumption); with the insecticides having the highest consumption (39%) rate 4. Recent years have also seen a growing trend towards the use of organophosphate (OP) pesticides, and these compounds are considered as one of the highly practical as well as frequently used pesticides among developing nations 5.

Only in Iran, it is estimated that the third cause of poisonings in general and first cause of deaths from poisonings are related to the OP pesticides use or exposure^6^. Organophosphate compounds due to their lipophilic structure and hence dermal absorption can affect various systems and organs of the human body. In several studies, exposure to these compounds have been shown to cause impairments and dysfunctions of several organs such as cardiovascular, liver and kidney along with nervous, respiratory, hematologic, immune, metabolic pathways, and endocrine systems. Furthermore, a significant relationship between the exposure to the OP pesticides and the mutagenicity and carcinogenicity of these compounds has been reported 7–9.

Formulators, farmers, gardeners, sprayers, and researchers are the occupational groups who might be exposed to various types of pesticides 10, 11. In the work environments, inhalation is the most frequent route of chemical exposure^12^–^14^ and as mentioned in the literature, the economic burden and financial cost of occupationl respiratory diseases is enormous 15. Almost similarly, for occupational exposure to OP pesticides, the inhalational and dermal routes are strongly predominant 16, 17. For instance, in the study by Damalas et al. on farmers, skin exposure was reported to be the primary route of exposure to pesticides^18^. The lipophilic nature of these compounds has led to their easily penetration and absorption 19; So that the effects such as systematic inflammation or sensitization from skin exposure has been reported as well 17. On the other hand, as Han stated, the inhalation exposure to airborne concentrations of OP pesticides are the principal route of exposure for formulation workers of these compounds 20.

In the pesticide formulation industry, where active ingredients are mixed with solvents, boosters, and fillers to achieve the desired formulation, many workers/formulators are routinely exposed to the fine mists, dusts or fumigants mostly by inhaling such contaminants. These toxicants are mainly absorbed through the respiratory route and may eventually find their way into the bloodstream and impair the respiratory and hematologic systems 21. The critical adverse respiratory health effects associated with the excessive exposure to higher concentrations of OP insecticides include: bronchoconstriction, pulmonary edema, mucous membrane secretions of the lung and paralysis of the respiratory muscles 22.

Some researchers have reported that OP pesticides can cause injuries and damage to the liver and kidney tissues^8^,^23^. OP insecticides have also shown to be hematotoxic and contribute to anemia through interfering with iron metabolism and reducing its total binding capacity levels to hemoglobin^21^.

As pesticide formulation workers have routine, continuous, prolonged and excessive exposures as well as higher risk of developing sever adverse health effects and regarding the fact that only a few studies have tended to consider and investigate their occupational exposures^11^,^24^, the present study was designed to determine whether occupational exposure to OP pesticides is associated with any hematologic, liver, kidney, nervous and respiratory system dysfunctions and impairments among OP pesticides formulators.

## Methods

### Study design and population

This cross-sectional study was performed on male formulators working in an OP pesticide formulation plant during the peaks of their production. The study population consisted of 28 workers/formulators (as the exposed group) and 17 office employees of the same plant (as the non-exposed or control group). The sample size in both groups was chosen using the census method. According to this method, all participants, both workers/formulators and office employees, were eligible to enter the study and accepted the invitation from the researchers; particularly after being provided with necessary information about the study. All of the exposed workers had been actively working in the production line of the plant since at least six months ago (the minimum six months of work experience in the production line). Moreover, the office employees did not have any history or background of exposure to OP pesticides or other toxic chemicals.

In this study, for each person, a questionnaire containing demographic characteristics such as age, work experience, height, weight, etc., smoking status, job history, history of exposure to chemicals, record of any physical and mental illness, taking medications and drugs, etc. was completed. Also, the exposure and control groups were matched in terms of work experience, height, weight, body mass index (BMI) and smoking.

The inclusion criteria included: not suffering from diabetes, hematologic or respiratory diseases, no liver, kidney and psychoneurological disorders, no alcohol consumption, no history of chest or abdominal surgery and no record of taking medications such as antibiotics (amoxicillin, clonate, trimethoprim, sulfamethoxazole, azithromycin), analgesics and nonsteroidal anti-inflammatory drugs (NSAIDs) (ibuprofen, naproxen sodium, ketoprofen, acetaminophen, aspirin), statin (rosuvastatin, atorvastatin, and simvastatin), diuretics (hydrochlorothiazide and furosemide), cardiovascular (amiodarone, calcium channel blockers, and quinidine), antidepressants (duloxetine and tianeptine) and corticoid drugs^25^–^27^. The data required for inclusion of the subjects in the study was extracted and obtained through medical records and annual/periodic examinations, occupational medicine checkup, questionnaires, and interviews.

In this study, the measurement and monitoring were performed on the last working day and before the beginning of the work. So that, firstly, in the fasting state, blood and urine samples were taken, then the ATS-DLD and Q16 questionnaires were completed and finally spirometry test was performed. Production line and office workers work 8 hours a day, five days a week, and in this respect, the two groups are considered similar.

### Blood specimens

#### Blood sample collection

Being in the fasting state, an experienced operator collected 7ml of venous blood specimen from each participant. 2 ml of the specimen was then kept in the CBC non-vacuum tube (buffered K2 EDTA, 201001 catalog number, Farzaneh Arman Co., Iran) in order to implement hematological assays. Furthermore, the rest of the specimen was poured into GEL tube (gel and clot activator, XLPCGC85 catalog number, Xinle Co., China) to implement biochemical assays. In the GEL tube, the serum of the specimen was separated by 16-branch centrifuge device (Lab Trone co., Iran). The specimens were labeled and secured in ice packs and immediately transported to the laboratory where the CBC tubes were analyzed and the serum of specimens aliquoted and stored in -20 °C.

The main approaches of quality control (QC) and quality assurance (QA) in blood samples include: utilizing the technician on double-blind bases, using calibrator, control, and blank samples (like Trucal U, Trulab N, Trulab P, distilled water, and etc.) and comparing them with control curves and reference values according to the recommendations and guidelines of manufacturers, analyzing several samples by manual and instrumental methods and comparing their results, using the check test method, calibrating the tools and devices of analyzers, no exposure to sun, light, and heat, having the appropriate laboratory environment temperature, cleansing and disinfecting the laboratory, hence preventing the growth of bacteria, fungi, and etc.

### Hematological assays

The measurement of hematological parameters such as white blood cell (WBC) count and percentage of differential (WBC diff), red blood cell (RBC) count, hemoglobin (Hgb), hematocrit (HCT), mean corpuscular volume (MCV), mean cell hemoglobin (MCH), mean corpuscular hemoglobin concentration (MCHC), platelets (PLT), red blood cell distribution width (RDW), platelet distribution width (PDW), and mean platelet volume (MPV) were performed through fluorescence flow cytometry (FFC) method via an automated hematology analyzer (XS-800i model, Sysmex Co,. Japan).

### Biochemical assays

The measurement of biochemical parameters such as fasting blood sugar (FBS), total cholesterol (TC), high density lipoprotein cholesterol (HDL-C), low density lipoprotein cholesterol (LDL-C), creatinine (Cr), serum uric acid (SUA), blood urea nitrogen (BUN), aspartate aminotransferase (AST), alanine aminotransferase (ALT), direct bilirubin (DBIL), total bilirubin (TBIL), total protein (TP), and albumin (ALB) was performed through commercial diagnostic kits (Pars Azmoon Co,. Iran) via an auto analyzer (BT-3000 model, Biotechnical Co, Italy).

### Urine sample

Simultaneously with collecting the blood specimens from the participants, the first morning urine samples were collected in polyethylene containers (labeled with defined codes for each person). The samples were then placed inside a safety box which contained several ice packs and immediately transported to the laboratory where the urinalysis (visual/physical, chemical, and microscopic examination of urine) was performed. The visual test included the color and clarity/appearance and was examined by a laboratory technician. The chemical examinations were performed via refractometer (SU-202 model, ERMA Co, Japan) and special strips (matrix test strips of Macherey Nagel Co, Germany) by colorimetric method for determination and detection of specific gravity level, PH level, protein, glucose, ketone, bilirubin, urobilinogen, blood, yeasts, and nitrite in urine sample. A microscope (CX-22LED model, Olympus Co, Germany) was utilized for identification and examinations of WBC, RBC, epithelial cells, bacteria, casts, and crystals based on the manufacturer's protocol in urine sample 28.

It should be noted that QC/QA approaches of blood specimens were applied for urine samples. Additionally, other approaches such as keeping the urine strip in closed containers, dipping a sufficient amount of strip with a urine sample (neither too little nor too much), examining the strips under appropriate lighting, calibrating the microscope, etc. were also utilized.

### Questionnaires

#### American Thoracic Society-Division of Lung Diseases (ATS-DLD)

As one of the commonly used questionnaires in workplace, ATS-DLD questionnaire was used in order to determine the prevalence of respiratory symptoms and illnesses^29^. In several Iranian studies, a translated (Persian) version of this questionnaire with slight modifications (similar to the one used in the present study) have been employed 30, 31. For instance, in Neghab et al. study, the Cronbach's alpha coefficient of this questionnaire (Persian version) was achieved as 0.77 30. AST-DLD questionnaire contains questions regarding demographic information, respiratory symptoms (cough, phlegm, wheezing, shortness of breath, chest tightness), smoking habits and medical, occupational and family history 31. In the present questionnaire, zero and one codes were assigned to No and Yes responses, respectively.

#### Neurotoxic Symptoms Questionnaire (Q16)

The Q16 is one of the most important screening tools to determine the prevalence of neurotoxic symptoms among workers exposed to solvents, pesticides, etc.^32^,^33^. Similarly, the translated (Persian) version of this questionnaire has already been used in several Iranian studies^32^, ^34^. This questionnaire consists of 16 questions with Yes/No answers, so that Yes and No answers are scored with one and zero codes, respectively. The total score is the sum of the Yes answers (one code) to the questions. Therefore, workers with more than 6 positive answers (Yes answers) are considered as the ones with early neurobehavioral symptoms^35^.

### Spirometry

The portable calibrated spirometer (Spirobank II model, A23-0Y.09541 serial number, MIR Co., Italy) was used to conduct the pulmonary function test (PFTS). These tests were performed by a skillful technician in accordance with the American Thoracic Society (ATS) recommendations^36^. In the present study, the measured parameters included forced vital capacity (FVC), forced expiratory volume in one second (FEV1), ratio FEV1/FVC, peak expiratory flow (PEF), forced expiratory flow between 25 and 75% expired volumes (FEF25-75%). Workers were asked not to perform the following activities within the specified periods before the test begins: performing strenuous physical activities (30 min), smoking (1h), eating heavy meals (2 h), having a bath (2h). Furthermore, necessary trainings were provided for accurate implementation of the spirometry test 37. In a standing position, the participants height and weight were measured and after five minutes of rest (in a sitting position), spirometry test was performed (between three and eight acceptable maneuvers for each person). Finally, the mean of percentage predicted values of the measurement parameters based on age, gender, weight, height, smoking and race was calculated using a spirometer. In this study, the criteria for spirometric results were classified into four patterns: obstructive (FEV1<80% predicted value and ratio FEV1/FVC<75%), restrictive (FVC<80% predicted value), mixed (a combination of obstructive and restrictive patterns), and normal (FEV1>80%, FVC>80% predicted value, and ratio FEV1/FVC>75%) 26.

It should be noted that spirometry measurements were performed at the same hours for both groups during the two morning consecutive days and before starting the work; one day for the exposed group and one day for the control group. In other words, an attempt was made to conduct these measurements in similar conditions for both groups.

### Ethical consideration

The present study was approved by research ethics committee of the Tarbiat Modares University (IR.MODARES. REC.1397.258). Moreover, the Helsinki Declaration (Version 2013) was taken into account while performing the present study 38. Basic ethical considerations such as explaining and describing the objectives of the study, obtaining the informed consent, maintaining anonymity and confidentiality as well as respecting the right to leave the study by the subjects, were considered in this study.

### Data analysis

Data were analyzed using SPSS v.22. The Kolmogorov-Smirnov test was used to determine the normal distribution of dependent variables. Mean and standard deviation were applied to describe numerical variables, while frequency and percentage were applied to describe categorical variables. The independent sample T-test and Mann-Whitney U test were applied for comparison of the hematological, biochemical, and spirometric (actual and predicted values) parameters between the exposed and control groups. Besides, these tests were employed to determine the association between cigarette smoking and quantitative variables. The Chi-square or Fisher's exact tests were measured to compare the urinalysis parameters, respiratory (ATS-DLD) and neurobehavioral (Q16) symptoms between the exposed and control groups; in the same manner, these tests were applied to determine the association between cigarette smoking and qualitative variables. Fisher's exact test has been used when the expected value in one of the cells were less than 5. The Pearson and Spearman correlation tests were used to determine the relationship between work experience and other dependent variables. A p-value of less than 0.05 was considered statistically significant.

## Results

In this study, there was no statistically significant difference with regard to work experience, height, weight, BMI, and cigarette smoking between the exposed and the control groups (p>0.05). However, the age of the workers was significantly lower in the exposed group than in the control group (p<0.05). The information details about demographic characteristics are presented in Table 1.

**Table 1:** Demographic characteristics of the study population

Variable	Mean ± SD		P-value
	Exposed group (n= 28)	Control group (n= 17)	
Age (year)	37.4 ± 7.33	42.88 ± 8.04	0.024 a, *
Work experience (year)	13.23 ± 7.7	17.23 ± 7.33	0.093 a
Height (cm)	175.5 ± 8.37	174.7 ± 6.89	0.744 a
Weight (kg)	77 ± 13.4	77.4 ± 13.36	0.921 a
Body Mass Index (BMI) (Kg/m^2^)	24.98 ± 4.02	25.25 ± 3.65	0.825 a

	**Percentage**		
Cigarette smoking			
Yes	39.3 % (n= 11)	17.6 % (n= 3)	0.116 b
No	60.7 % (n= 17)	82.4 % (n= 17)	

SD: standard deviation

a Independent sample T-test

b Fisher's exact test

* P-Value<0.05

Based on the analysis of blood and urine samples, no statistically significant difference was observed between the mean hematological, biochemical and urinalysis parameters, except in specific gravity, between the exposed and the control groups (p>0.05). Comparisons of hematological, biochemical and urinalysis parameters between the exposed and the control groups are shown in Table 2.

**Table 2:** Analysis of blood and urine samples

	Variable	Mean ± SD		P-value
		Exposed group (n= 28)	Control group (n= 17)	
Hematological indices	WBC (10^3^/µL)	7.08 ± 1.65	6.64 ± 0.71	0.543 ^b^
	- Neutrophils (%)	48.63 ± 10.15	47.59 ± 4.96	0.652 ^a^
	- Lymphocytes (%)	39.5 ± 8.54	40.06 ± 4.53	0.78 ^a^
	- Monocytes (%)	8.52 ± 1.87	9.053 ± 1.85	0.366 ^a^
	- Eosinophils (%)	2.98 ± 1.98	2.96 ± 1.56	0.885 ^b^
	- Basophils (%)	0.36 ± 0.13	0.34 ± 0.17	0.738 ^b^
	RBC (10^6^/µL)	5.53 ± 0.48	5.23 ± 0.53	0.056 ^a^
	Hgb (g/dL)	15.86 ± 1.17	15.31 ± 1.01	0.121 ^a^
	HCT (%)	45.74 ± 3	44.43 ± 2.6	0.144 ^a^
	MCV (fL)	83.2 ± 6.9	85.5 ± 6.03	0.824 ^b^
	MCH (pg)	28.86 ± 2.66	29.46 ± 1.98	0.691 ^b^
	MCHC (g/dL)	34.67 ± 1.02	34.46 ± 0.76	0.483 ^a^
	PLT (103/µL)	237.14 ± 55.19	245.82 ± 61.17	0.626 ^a^
	RDW (%)	13.21 ± 1.14	13.06 ± 0.66	0.851 ^b^
	PDW (fL)	13.42 ± 1.61	13.08 ± 1.72	0.518 ^a^
	MPV (fL)	10.7 ± 0.8	10.54 ± 0.73	0.509 ^a^

Liver Function Tests (LFTs)	AST (U/L)	27.96 ± 7.21	29 ± 7.13	0.452 ^b^
	ALT (U/L)	27.53 ± 12.63	27.06 ± 13.98	0.833 ^b^
	DBIL (mg/dL)	0.41 ± 0.02	0.2 ± 0.01	0.101 ^b^
	TBIL (mg/dL)	0.48 ± 0.34	0.47 ± 0.26	0.953 ^a^
	TP (g/dL)	7.01 ± 1.27	7.02 ± 1.36	0.756 ^b^
	ALB (g/dL)	6.82 ± 2.13	5.63 ± 1.9	0.079 ^a^

Kidney Function Test (KFTs)	Cr (mg/dL)	0.56 ± 0.04	0.47 ± 0.02	0.796 ^b^
	SUA (mg/dL)	6.95 ± 1.98	7.15 ± 3.12	0.882 ^b^
	BUN (mg/dL)	31.41 ± 6.2	34.4 ± 5.8	0.131 ^a^

Urinalysis (Continuation of kidney function tests)	Color (% Yellow)	100	100	-
	Appearance (% Clear)	88.9	94.1	0.496 ^c^
	Specific gravity	1.0225 ± 0.003	1.02018 ± 0.003	0.025 *, ^b^
	PH	5.03 ± 0.2	5.12 ± 0.48	0.713 ^b^
	Protein (% Negative)	96.3	100	0.614 ^c^
	Glucose (% Negative)	92.6	100	0.371 ^c^
	Ketone (% Negative)	100	100	-
	Bilirubin (% Negative)	100	100	-
	Urobilinogen (% Negative)	100	100	-
	Blood (% Negative)	96.3	100	0.614 ^c^
	Yeasts (% Negative)	100	100	-
	Nitrite (% Negative)	100	100	-
	Others (% Negative)	100	100	-
	WBC (0–2/hpf) (%)	92.6	94.1	0.671 ^c^
	RBC (0–2/hpf) (%)	96.3	100	0.614 ^c^
	Epithelial Cells (% Negative)	96.3	100	0.614 ^c^
	Bacteria (% Negative)	96.3	100	0.614 ^c^
	Mucus (% Negative)	96.3	100	0.614 ^c^
	Casts (% Negative)	96.3	100	0.614 ^c^
	Crystals (% Negative)	92.6	88.2	0.504 ^c^

**lipid profile**	TC (mg/dL)	159.43 ± 27.22	152.35 ± 28.02	0.408 ^a^
	HDL-C (mg/dL)	34.23 ± 11.5	36.23 ± 13.7	0.62 ^a^
	LDL-C (mg/dL)	88.07 ± 30.17	83.87 ± 34.36	0.685 ^a^
	FBS (mg/dL)	93.53 ± 28.45	88.53 ± 16.38	0.888 ^b^

**Figure 1:**
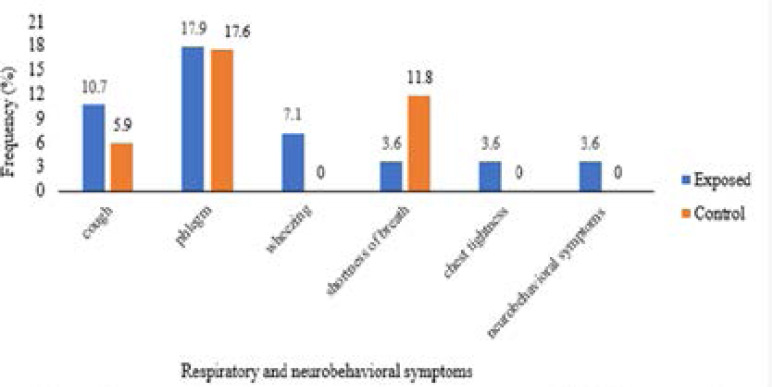
Frequency of respiratory and neurobehavioral symptoms (p>0.05) (Fisher's exact test)

No statistically significant association was found for the respiratory and neurobehavioral symptoms parameters between the exposed and the control groups (p>0.05). Moreover, the prevalence of respiratory and neurobehavioral symptoms was 10.7 % for cough, 17.9 % for phlegm, 7.1 % for wheezing, 3.6 % for shortness of breath, chest tightness, and neurobehavioral symptoms among the exposed group (Table 3 and Figure 1).

**Table 3:** Frequency of Yes/No answer to respiratory and neurobehavioral symptoms

			Exposed group (n= 28)	Control group (n= 17)	P-value
Respiratory symptoms		Answer	Percentage (%)	Percentage (%)	
	cough	Yes	10.7	5.9	1.00 a
		No	89.3	94.1	
	phlegm	Yes	17.9	17.6	1.00 a
		No	82.1	82.4	
	wheezing	Yes	7.1	0	0.519 a
		No	92.9	100	
	shortness of breath	Yes	3.6	11.8	0.547 a
		No	96.4	88.2	
	chest tightness	Yes	3.6	0	0.622 a
		No	96.4	100	
	neurobehavioral	Yes	3.6	0	0.622 a

Spirometry findings did not show a significant difference in the mean FVC, FEV1, FEV1/FVC, PEF, and FEF25-75% parameters between the exposed and the control groups (p>0.05). Moreover, the frequency of abnormal spirometry pattern for the exposed and the control groups were 15.4 % and 8.3 %, respectively (p>0.05). Comparisons of spirometric indices and patterns between the exposed and the control groups are shown in Table 4.

**Table 4:** Spirometric indices and patterns

	Variable	Mean ± SD		P- value c
Spirometric indices		Exposed group (n= 28)	Control group (n= 17)	
	FVC a (L)	4.86 ± 1.03	4.82 ± 1.02	0.914
	FEV1 a (L)	3.85 ± 0.87	3.66 ± 0.59	0.51
	FEV1/FVC a (%)	78.8 ± 5.9	76.7 ± 4.7	0.285
	PEF a (L/S)	10.01 ± 2.04	9.65 ± 1.32	0.586
	FEF25–75% a (L/S)	3.83 ± 1.33	3.22 ± 0.61	0.062
	FVC b (%)	99.8 ± 14.26	103.9 ± 14.55	0.418
	FEV1 b (%)	95 ± 15.27	96.5 ± 9.48	0.757
	FEV1/FVC b (%)	94.96 ± 6.76	93.08 ± 5.7	0.41
	PEF b (%)	109.34 ± 21.23	108.75 ± 11.32	0.911
	FEF25–75% b(%)	85.7 ± 27.15	80.6 ± 12.45	0.432

Spirometric patterns	obstructive pattern (%)	7.7	8.3	-
	restrictive pattern (%)	3.8	0	-
	mixed pattern (%)	3.8	0	-
	normal pattern (%)	84.6	91.7	-
	abnormal pattern (%)	15.4	8.3	0.488 d

FVC: forced vital capacity, FEV1: forced expiratory volume in one second, PEF: peak expiratory flow, FEF_25–75_%: forced expiratory flow between 25 and 75% expired volumes

a the mean of actual values

b the mean of percentage predicted values, SD: standard deviation

c Independent sample T-test

d Fisher's exact tests

* P-Value<0.05.

Among the participants of the exposed group, the mean spirometric parameters (FVC, FEV1, FEV1/FVC, PEF, and FEF25-75%) were significantly lower for smokers (P<0.05). The details of the association between work experience and smoking with dependent variables in the exposed group are described in Table 5.

**Table 5:** Association between work experience and cigarette smoking with dependent variables in exposed group

	Variable	Work experience h (n=28)		Smoker (n= 11)	Smoking non- smoker (n= 17)	
		r	P	Mean ± SD	Mean ± SD	P
Hematological indices	WBC (10^3^/µL)	0.401 b	0.034 *	7.6 ± 1.95	6.75 ± 1.4	0.151 d
	- Neutrophils (%)	0.275 c	0.165	46.8 ± 10.67	49.7 ± 10	0.481 e
	- Lymphocytes (%)	- 0.298 c	0.131	40.82 ± 9.57	38.73 ± 8.08	0.55 e
	- Monocytes (%)	- 0.115 c	0.569	8.51 ± 1.8	8.53 ± 1.97	0.974 e
	- Eosinophils (%)	0.075 b	0.708	3.53 ± 1.83	2.66 ± 2.11	0.175 d
	- Basophils (%)	- 0.367 b	0.060	0.35 ± 0.09	0.37 ± 0.16	0.836 d
	RBC (10^6^/µL)	0.218 c	0.265	5.44 ± 0.25	5.6 ± 0.58	0.377 e
	Hgb (g/dL)	- 0.061 c	0.757	16.34 ± 0.9	15.47 ± 1.25	0.079 e
	HCT (%)	0.082 c	0.68	46.95 ± 2.76	44.97 ± 2.96	0.088 e
	MCV (fL)	0.131 b	0.506	86.3 ± 3.04	81.2 ± 8	0.029 *,^d^
	MCH (pg)	- 0.011 b	0.954	30.05 ± 0.81	28.09 ± 3.15	0.015 *, d
	MCHC (g/dL	- 0.351 c	0.067	34.85 ± 1.04	34.54 ± 1.02	0.447 e
	PLT (10^3^/µL)	0.128 c	0.515	224.63 ± 53.8	245.23 ± 56.15	0.344 e
	RDW (%)	0.546 b	0.003 *	12.95 ± 0.5	13.39 ± 1.4	0.378 d
	PDW (fL)	0.244 c	0.229	13.66 ± 1.9	13.27 ± 1.44	0.559 e
	MPV (fL)	0.228 c	0.264	10.82 ± 0.92	10.63 ± 0.73	0.581 e

Liver Function Tests (LFTs)	AST (U/L)	0.103 b	0.601	28.18 ± 10.17	27.82 ± 4.8	0.404 d
	ALT (U/L)	0.064 b	0.746	23.09 ± 11.03	30.4 ± 13.1	0.073 d
	DBIL (mg/dL)	- 0.180 b	0.378	0.36 ± 0.04	0.45 ±0.05	0.737 d
	TBIL (mg/dL)	0.073 c	0.722	0.43 ± 0.32	0.5 ± 0.4	0.613 e
	TP (g/dL)	- 0.361 b	0.07	6.67 ± 0.64	7.37 ± 1.5	0.201 d
	ALB (g/dL)	0.099 c	0.63	6.44 ± 2.14	7.07 ± 2.15	0.477 e

Kidney Function Test (KFTs)	Cr (mg/dL)	0.339 b	0.09	0.65 ± 0.05	0.49 ± 0.02	0.856 d
	SUA (mg/dL)	0.291 b	0.15	6.6 ± 2.28	7.16 ± 1.8	0.551 d
	BUN (mg/dL)	0.017 c	0.936	30.09 ± 4.93	32.25 ± 6.9	0.4 e

Urinalysis (Continuation of kidney function tests)	Appearance	0.084 b	0.678	90 g	80 g	0.697 f
	Specific gravity	- 0.389 b	0.045 *	1.02160 ± 0.003	1.02300 ± 0.002	0.346 d
	PH	0.165 b	0.411	5 ± 0.2	5.06 ± 0.24	0.443 d
	Protein	0.165 b	0.411	100 g	94.1 g	0.63 f
	Glucose	0.302 b	0.126	90 g	94.1 g	0.613 f
	Blood	0.165 b	0.411	100 g	94.1 g	0.63 f
	WBC	- 0.009 b	0.964	90 g	94.1 g	0.613 f
	RBC	0.165 b	0.411	100 g	94.1 g	0.63 f
	Epithelial Cells	0.165 b	0.411	100 g	94.1 g	0.63 f
	Bacteria	0.165 b	0.411	100 g	94.1 g	0.63 f
	Mucus	- 0.051 b	0.802	90 g	100 g	0.37 f
	Casts	0.165 b	0.411	100 g	94.1 g	0.63 f
	Crystals	- 0.018 b	0.928	90 g	94.1 g	0.613 f

Lipid profile	TC (mg/dL)	0.147 c	0.455	156.27 ± 21.32	161.47 ± 30.9	0.613 e
	HDL-C (mg/dL)	0.116 c	0.573	36.13 ± 12.03	33.04 ± 11.43	0.517 e
	LDL-C (mg/dL)	0.256 c	0.207	83.78 ± 25.8	90.78 ± 33.12	0.575 e
	FBS (mg/dL)	0.402 b	0.034 *	105.36 ± 41.97	85.88 ± 10.3	0.134 d

Spirometric indices	FVC a (%)	- 0.140 c	0.494	92.4 ± 13.62	104.44 ± 12.98	0.033 *, e
	FEV1 a (%)	- 0.246 c	0.226	85.2 ± 15.9	101.125 ± 11.5	0.007 *, e
	FEV1/FVC a (%)	- 0.362 c	0.069	91.5 ± 6.5	97.125 ± 6.14	0.036 *, e
	PEF a (%)	- 0.128 c	0.532	96.8 ± 20.8	117.19 ± 17.9	0.014 *, e
	FEF25–75% a (%)	- 0.276 c	0.173	69.6 ± 23.55	95.75 ± 24.8	0.014 *, e

Respiratory symptoms	Cough (%)	0.079 b	0.688	27.3	0	0.05 f
	Phlegm (%)	0.047 b	0.814	27.3	11.8	0.29 f
	Wheezing (%)	0.294 b	0.128	18.2	0	0.146 f
	shortness of breath (%)	0.252 b	0.195	9.1	0	0.393 f
	chest tightness (%)	0.252 b	0.195	9.1	0	0.393 f
	neurobehavioral symptoms (%)	- 0.06 b	0.761	9.1	0	0.393 f

r: Correlation

a The mean of percentage predicted values

b *Spearman's* correlation coefficient

c Pearson's correlation coefficient

d Mann–Whitney U

e Independent sample T-test

f Fisher's exact tests

g % Negative

h Work experience is defined as the number of years that a person has been exposed to organophosphate pesticides

* P-Value<0.05.

## Discussion

In the workplace, disorders and adverse health effects caused by exposure to pesticides are ever-increasing. Researchers have reported that the prevalence of pesticide-induced diseases in the occupational settings are 1.17 per 100,000 whole time equivalent employees 17. Therefore, the harmful effects of pesticides on human health are remained as an alarming concern.

In the present study, no significant difference was found for the hematological, biochemical, urinalysis, respiratory, and neurobehavioral indices between the exposed and the control groups. Moreover, these indices were in the normal range. Therefore, as the results indicated, the male OP pesticide workers/formulators were not found to be suffering from disorders and diseases induced by exposure to such pesticides. The possible explanations for the current results might be that 1. In different units of the production line, the local exhaust ventilation (LEV) system including downdraft or canopy hoods, ducts, and centrifugal fans have been designed and installed near the source of pollution. In some units, such as the powder production unit, a cyclone has been added to the LEV system as a cleaning system. Therefore, the design and implementation of this ventilation system not only limits and reduces the workers' exposure to organophosphate pesticides on the production line, but also prevents its possible release and emission into the outdoor environment;^2^. Adequate personal protection through the proper use of personal protective equipment (PPE) such as chemical-resistant work attire, apron, footwear and gloves along with neoprene rubber or barrier laminate models and respiratory protection masks such as FFP3 or FFP2 types had been provided for the workers/formulators working in the production line. It should be noted that workers and formulators' clothes are washed at the end of each work shift; 3. The workers/formulators of the production line do not work in a fixed position or wok station. In this factory, with in each month, the production line workers exchange their jobs rotationally among different units such as formulation, powder production unit, packaging, etc; 4. The plant has a high ceiling leading to minimal accumulation of the pollutant and hence less inhalation and skin exposure; 5. The type, the level of toxicity and the potency of pesticides are the major factors affecting the human health. In fact, the studied plant produces four types of OP pesticides: Diazinon, Chlorpyrifos, Malathion, and Ethion. In the structure of these pesticides, there is a double bond phosphorus-sulfur (Phosphorothioate and Phosphorodithioate groups) which makes the toxic response of these compounds less potent 12. In view of all that has been mentioned so far, we may suppose that exposure and toxic response to OP pesticides are not particularly considerable among the production line workers/formulators in the present study. The previous studies have also considered the health impact of pesticide exposure on the individuals.

Engineering controls adopted within the plant prevented the release of pesticides to the outside environment. In addition, engineers, and those who checked the production line from time to time, used PPE as well. No considerable symptoms of poisoning and occupational disease were observed and reported in the documentation of Health, Safety and Environment (HSE) management unit of this factory. Based on the documentation and annual air monitoring/quality performed by the HSE unit, no organophosphate pesticides were found in the administrative unit. It should also be noted that the distance between the production line and the office unit is approximately 500 meters.

Stacey express that the prevalence of damage to the hematopoietic system due to exposure to chemicals compounds such as pesticides is not significant 12. Likewise, in the present study, no significant hematological changes were observed among both the exposed and the control groups. Our findings are also relatively consistent with several earlier research by Miranda Adad et al. 39, Aroonvilairat et al. 40, Al-Sarar et al. 41, Abeer Arafa et al. 42, Piccoli et al. 43, Contreras et al. 44, and Remor et al.^45^. Nonetheless, previous research findings into adverse health effects of exposure to pesticides have been inconsistent and contradictory. For instance, while studies by Garcia et al. 46, Quraishi et al. 47, and Manyilizu et al. 48 have reported a significant increase and exacerbation of the symptoms; Neghab et al. 28, Cortes-Iza et al. 49, Cestonaro et al. 50, and Cattelana et al. 51 have shown a significant decrease. The discrepancy among the results can be due to factors such as: pesticide type, exposure conditions (concentration of pesticides, duration of exposure), geographical contexts, using personal protective equipment among workers, methodology as well as the employed statistical tests. As a result, the possible role of OP pesticides exposure in inducing the hematological symptoms requires extensive and further investigations and there is no definite and certain evidence in this regard.

The liver and kidneys are the two main organs in the human body that perform detoxification, biotransformation, elimination, and excretion process of toxic chemicals such as pesticides; therefore, pesticides are capable of impairing these target organs 52. In our study, liver and kidney injuries from exposure to OP pesticides were not observed. Also referring to the previous studies, a significant difference in liver and kidney indices from Al-Sarar et al. 41 and Neghab et al. ADDIN EN.CITE Neghab201841^28^ 414117Neghab, MasoudJalilian, Hamed-Taheri, ShekoufehTatar, MohsenZadeh, Zeynab Haji-Life sciencesLife sciences182-18720220180024-3205 10.1016/j.lfs.2018.04.020 28 studies, as well as liver enzymes from the study by Aroonvilairat et al. 40 were not observed. Manfo et al. 52 indicated that pesticides have no effect on the AST enzyme, uric acid, and creatinine. In the studies by Contreras et al. 44 and Miranda Adad et al. 39, there was no significant difference in biochemical parameters such as liver enzymes, glucose, creatinine, uric acid, and lipid profile. The study by Ahmed Khan et al. 1 on workers of the pesticide formulation industry did not indicate any significant changes in the levels of parameters such as bilirubin, albumin, total protein, urea, and creatinine. Garcia et al. 46, Singh et al. 53, Yasser El-Nahal^54^, and Cestonaro et al. 50 showed that pesticids impair liver and kidney indices and lipid profile. It is worth noting that in the above-mentioned studies, the study population were the farmers and pesticide sprayers who were mostly exposed to a mixture of several pesticides simultaneously. In these occupational groups, exposure to pesticides is seasonal and highly acute; also, these workers are exposed to other pollutants such as dusts, gases, bioaerosols, endotoxins and fertilizers; whereas, the factory workers are not routinely exposed to these other pollutants.

In the present study, there was no significant difference in spirometric parameters and respiratory and neurobehavioral symptoms between the exposed and the control groups; however, for the exposed group, such parameters showed a significant decrease among the smokers rather than non-smokers. Similar to the findings of the present study, Quansah et al. 55, Ngowi et al. 56, and Jones et al. 57 showed that exposure to OP pesticides did not affect spirometric parameters and induced no respiratory symptoms. In the study by Neghab et al. 58 conducted on workers of a plant that produced organochlorine, organophosphate, carbamate, and paraquat pesticides, a significandecrease in spirometric parameters and a significant increase in the severity of the respiratory symptoms were found between the exposed and the control groups. However, the results from the present study was only attributed the plant that only produced OP pesticides. In the studies by Woldeamanuel et al. 15 and Atkure Defar and Ahmed Ali 59, there was a significant difference in spirometry indices and respiratory symptoms. The main reason for the difference between the results of the present study and the above two studies related to the study population. Woldeamanuel et al. study on farmers and Atkure Defar and Ahmed Ali study on greenhouse workers were performed. In addition, greenhouse workers are exposed to 127 different chemicals and have a high chance in respiratory disorders and diseases. In the studies by Jones et al. 57 and Abu Sham'a et al. 60, similar to the present study, smoking was identified as a parameter affecting on lung function. The destructive substances in cigarettes (Cyanide, Nicotine, and Thiocyanate) can cause these effects^11^. Thus, the workers of smokers than non-smokers are higher chances and probability in lung dysfunction.

In a study by Starks et al. 61, similar to the present study, the results from the neurotoxic tests showed that a significant association between exposure to OP pesticides and neurobehavioral parameters were not observed. Zaidi et al. 62 reported that significant changes in some neurobehavioral parameters were found among factory workers. The major causes for the difference between such results and the present study might be related to the type of pesticides produced in this plant, the type of tests used to determine neurobehavioral symptoms and the location of conducting the study. While Zaidi et al. had studied the health effects of Triazophos and Acephate insecticides, in our study, Diazinon, Chlorpyrifos, Malathion, and Ethion insecticides were the major formulated pesticides.

Finally, one of the principal limitations of the present study that should be mentioned here is that the air sampling and measurement of the airborne pollutants which were particularly present in the work environment and breathing zones of the workers/formulators were not performed. This was of course due to the fact that permission for conducting such measurement was not granted by the plant manager. Another limitation of this study is that workers are exposed to a combination/mixture of several organophosphate pesticides, and it is difficult to generalize/ contribute the effects to a specific pesticide. Moreover, because the present study was cross-sectional, it is difficult to establish convincing evidence of a causal relation between exposure to organophosphate pesticides and harmful effects/ absence of harmful effects, and therefore the results of this study suggest this relationship.

## Conclusion

Based on the working conditions and the results from the present study, exposure to OP pesticides did not contribute to any adverse health effects among the male workers/formulators and these workers did not show any indications of OP-induced disorders and diseases. Nevertheless, higher risk of developing respiratory dysfunctions and disorders were observed among smoker subjects (compared to non-smokers). The authors would emphasize that decision making over the health risks of workers/formulators of the OP pesticide formulation industry requires further comprehensive studies, and in particular, larger study populations should be taken into account.
